# Anti-Obesity Potential through Regulation of Carbohydrate Uptake and Gene Expression in Intestinal Epithelial Cells by the Probiotic *Lactiplantibacillus plantarum* MGEL20154 from Fermented Food

**DOI:** 10.4014/jmb.2212.12005

**Published:** 2023-02-06

**Authors:** So Young Park, Jin Won Choi, Dong Nyoung Oh, Eun Ji Lee, Dong Pil Kim, Sun Jay Yoon, Won Je Jang, Sang Jun Han, Seungjun Lee, Jong Min Lee

**Affiliations:** 1 Department of Biotechnology, Pukyong National University, Busan 48513, Republic of Korea; 2 Department of Food Science and Nutrition, Pukyong National University, Busan 48513, Republic of Korea

**Keywords:** *Lactiplantibacillus plantarum* MGEL20154, anti-obesity, *erk2*, *pparα*, *glut2*

## Abstract

We investigated the probiotic characteristics and anti-obesity effect of *Lactiplantibacillus plantarum* MGEL20154, a strain that possesses excellent intestinal adhesion and viability. The in vitro properties, *e.g.*, gastrointestinal (GI) resistance, adhesion, and enzyme activity, demonstrated that MGEL20154 is a potential probiotic candidate. Oral administration of MGEL20154 to diet-induced obese C57BL/6J mice for 8 weeks resulted in a feed efficacy decrease by 44.7% compared to that of the high-fat diet (HFD) group. The reduction rate of weight gain was about 48.5% in the HFD+MGEL20154 group compared to that of the HFD group after 8 weeks, and the epididymal fat pad was also reduced in size by 25.2%. In addition, the upregulation of the *zo-1*, *pparα*, and *erk2*, and downregulation of the nf-κb and *glut2* genes in Caco-2 cells by MGEL20154 were observed. Therefore, we propose that the anti-obesity effect of the strain is exerted by inhibiting carbohydrate absorption and regulating gene expression in the intestine.

## Introduction

The nomadic probiotic *Lactiplantibacillus plantarum* is generally derived from fermented food and adapts to ecosystems, such as the gut, oral cavity, and vagina, that allow it to persist for at least a limited time due to its wide-ranging and flexible genomic properties. These attributes correspond to improved metabolic flexibility, conditional respiration, sugar uptake, and gastric and bile acid resistance [1, 2]. In particular, a well-defined benefit of *Lp. plantarum* in the human GI tract is regulating weight gain. Numerous clinical trials have confirmed the successful use of diverse *Lp. plantarum* strains as a dietary intervention to prevent and/or ameliorate obesity [3, 4]. Furthermore, they have been reported to inhibit differentiation and lipid accumulation in adipocytes and suppress obesity-induced inflammation by regulating the transcription of fatty acid synthase (FAS), CCAAT-enhancer-binding protein α (C/EBPα), peroxisome proliferator-activated receptor γ (PPARγ), leptin, AMP-activated protein kinase (AMPK) signaling pathways, janus kinase/signal transducer and activator of transcription proteins (JAK/STAT), and nuclear factor kappa-light-chain-enhancer of activated B cells (NF-κB) [5-9].

To maximize their health benefits, probiotics should be able to adhere to the intestinal epithelial layer, an ability which is one of the most important prerequisites for probiotics. In general, bacterial adhesion to the GI tract surface is a specific interaction between intestinal surface proteins and the membrane components of *Lp. plantarum*, such as alfa-enolase-1 (EnoA1), collagen binding protein (Cbp), elongation factor Tu (EF-Tu), flagellin protein (FliC), glyceraldehyde-3-phosphate dehydrogenase (GAPDH), and mannose-specific adhesion (Msa) protein [10-14]. However, trypsinization of *Lp. plantarum* did not completely eliminate adhesion capacity, suggesting that non-protein interactions involve non-specific mechanisms, such as hydrophobicity and electron donor-acceptor of bacterial cell wall, which significantly contribute to the initial adhesion interactions of *Lp. plantarum* [15]. Moreover, it has been reported that a significant positive correlation was revealed between bacterial cell surface hydrophobicity and adhesion capacity [16].

Therefore, this study was conducted to investigate the health-promoting effect of *Lp. plantarum*, a species derived from traditional fermented food which has excellent cell surface hydrophobicity and can directly adhere to Caco-2 cells. Here, we report that the *Lp. plantarum* strain MGEL20154 has strong adhesion abilities, maintains barrier integrity, regulates metabolic, immune, and cell differentiation-related gene expression, and alleviates fat accumulation. These novel observations provide insight into the rationale for using *Lp. plantarum* MGEL20154 as a potential alternative therapeutic for the palliative treatment of obesity. Taken together, our results suggest that *Lp. plantarum* MGEL20154 is an important candidate strain for the prevention and alleviation of metabolic diseases.

## Materials and Methods

### Bacterial Strains and Culture Conditions

A total of 18 bacterial strains, including 14 lactic acid bacteria (LAB) and four indicator strains, were used in this study: *Lactobacillus acidophilus* ATCC 4356^T^ (LA), *Lacticaseibacillus casei* ATCC 393 (LC), *Limosilactobacillus fermentum* KCTC 13097^T^ (LF), *Lactiplantibacillus plantarum* DSM 20174^T^ (LP), *Lacticaseibacillus rhamnosus* ATCC 53103 (strain GG; LR), *Lp. plantarum* MGEL20154 (MGEL20154), *Lp. plantarum* MGEL21083, *Lp. plantarum* MGEL21111, *Lp. plantarum* MGEL21118, *Lp. plantarum* MGEL21143, *Lp. plantarum* MGEL21144, *Lp. plantarum* MGEL21146, *Lp. plantarum* MGEL21155, *Lp. plantarum* MGEL21186, *Vibrio parahaemolyticus* ATCC 33844^T^, *Shigella sonnei* MGEL20007, *Listeria monocytogenes* KCTC 13064^T^, and *Bacillus cereus* KCTC 3624^T^. Each LAB or indicator strain was cultured overnight in De Man, Rogosa and Sharpe (MRS) or Brain Heart Infusion (BHI) media (Difco; Becton, Dickinson and Co., USA) under appropriate conditions (Table 1).

### Isolation and Identification of *Lp. plantarum*

Each strain of *Lp. plantarum* was isolated from homemade kimchi using MRS medium. All samples were homogenized with sterile phosphate-buffered saline (PBS, pH 6.5 ± 0.1). A 0.1 ml of serial diluted samples was spread-plated onto MRS agar containing 1% CaCO_3_. Each colony forming a clear zone was selected. The isolates were identified by 16S rRNA sequencing (Macrogen Inc., Korea). The sequences were compared and evaluated with available 16S rRNA sequences in the EzBioCloud server (CJ Bioscience Inc., Korea). A phylogenetic tree was constructed by the neighbor-joining method using the Kimura two-parameter model with MEGA7 software (RRID:SCR_000667) [17]. Morphological examination was performed by scanning electron microscopy (SEM; JSM-6490LV, Jeol, Japan) with Gatan mono CL3+ (Gatan, USA) at a voltage of 10 kV using cells grown for 18 h at 37°C.

### Cell Culture and Viability

Human epithelial Caco-2 cells (KCLB 30037, RRID:CVCL 0025) were maintained at 37°C under a humidified atmosphere of 5% CO_2_ in minimal essential medium (MEM; Sigma-Aldrich, USA) supplemented with 25 mM HEPES, 10% heat-inactivated fetal bovine serum, 1% non-essential amino acids, and 1% penicillin/streptomycin [18]. For passaging, confluent monolayers were sub-cultured every four days by treatment with 0.25% trypsin and 0.2% EDTA in PBS. The cells were then centrifuged at 1,000 ×*g* for 5 min, and ~10^6^ cells were resuspended with 8 ml of MEM and seeded into a new flask. Cells from passages 8–10 were used for all experiments. Cytotoxicity was investigated using the Cell Proliferation Assay Kit I (MTT based) (Sigma-Aldrich) according to the manufacturer’s protocol.

### Cell Surface Hydrophobicity, Auto- and Coaggregation

Bacterial surface hydrophobicity and autoaggregation were performed as described previously [19]. The values of adhesion to hexadecane for estimating hydrophobicity were calculated as follows in Eq. (1):

$$\text{hydrophobicity \%=}\left(\frac{{\text{A}}_{\text{0}}{\text{-A}}_{\text{t}}}{{\text{A}}_{\text{0}}}\right)\text{×100.}$$ (1)

A_0_ represents absorbance of the aqueous phase at 600 nm and A_t_ is the absorbance at 600 nm of the non-aqueous phase after 1 h at room temperature. The percentage of autoaggregations is expressed in Eq. (1), where A_t_ represents the absorbance at time (t) = 1 h and A_0_ is the absorbance at t = 0 h. The coaggregation assay was performed using a modified version of the method described by Nagaoka *et al*. (2008) [20]. Cultured bacterial cells were harvested by centrifugation at 4,000 ×*g* for 10 min and washed twice in PBS. Each cell pellet was resuspended in PBS to yield an OD_600_ (nm) of 1.0. Equal volumes (0.5 ml) of the pathogen and test strain cell suspensions were mixed in a cuvette, and the OD_600_ was measured immediately (A_0_). After incubation at room temperature for 1 h, the OD_600_ was again measured (A_t_). The percentage (%) of coaggregation was calculated using Eq. (1).

### Enumeration of Bacterial Cells Adhered to Caco-2

An overnight culture of each bacterial cell (~10^6^ colony forming units [CFU]/ml) was inoculated to monolayers of Caco-2 cells in 48‐well microtiter plates. The plates were incubated for 3 h at 37°C in a 5% CO_2_. Thereafter, the cells were washed three times with PBS. The adhered cells were treated with 1 ml of 0.5% Triton X-100 for 3 min on ice, and then the serially diluted samples with PBS were spread onto MRS agar to determine the adhered cells by direct counting [19].

### Tolerance during the Simulated GI Tract

The acid and bile tolerance were performed as previously described with minor modifications [21]. For simulating the saliva phase, each bacterial cell (~10^9^ CFU/ml) was suspended with an artificial saliva solution (30.0 g/l NaHCO_3_, 14.0 g/l, KCl, 4.0 g/l CaCl_2_, and 2.0 g/l NaCl) to a final volume of 20 ml.

For simulated gastric condition, the mixtures were acidified to pH 2 ± 0.2 with 1 ml of porcine pepsin preparation (0.04 g pepsin in 0.1 mol/l HCl). The samples were incubated in a shaking water bath at 85 rpm for 3 h at 37°C. After the gastric phase, the pH was increased to 5.0 ± 0.2 with 0.9 mol/l Na-bicarbonate, and then 200 μl of bile salts glycodeoxycholate (0.04 g in 1 ml of PBS), taurodeoxy-cholate (0.025 g in 1 ml of PBS), taurocholate (0.04 g in 1 ml of PBS), and 100 μl of pancreatin (0.04 g in 500 ml PBS) were added. The pH of each sample was increased to pH 7.5 ± 0.2 with 1 mol/l NaOH and the samples were incubated at 37°C in a shaking water bath at 85 rpm for 8 h to complete the simulated intestinal phase of the in vitro digestion process. Thereafter, appropriate dilutions were plated directly onto an MRS plate and incubated for 18 h at 37°C in 5% CO_2_ to determine the log10 CFU/ml.

### α-Amylase and Lipase Inhibition Assay

α-Amylase and lipase inhibition were performed using bacterial extracts as previously described with minor modifications [23, 24]. The cell extracts were prepared by ultrasonication using bacterial cells (~10^9^ CFU/ml) grown in MRS supplemented with 1% lactose for 16 h at 37°C under 5% CO_2_. After ultrasonication, bacterial cell debris was discarded, and the cell extract was freeze-dried. The dried cell extracts were dissolved in 10 mg/ml in 50 mM Na-acetate buffer (pH 6.5). For the α-amylase inhibition assay, 50 μg of α-amylase from porcine pancreatic (Sigma-Aldrich) was dissolved in a buffer consisting of 50 mM Na-acetate buffer (pH 6.5). Then, 20 μl of bacteria extract and 50 μl of 5 mM *p*-nitrophenyl-α-d-maltoside (pNPM) were added to the enzyme solution. For lipase inhibition assay, 50 μg of porcine pancreatic lipase (Sigma-Aldrich) was dissolved in a buffer consisting of 50 mM Na-acetate buffer (pH 6.5). Following that, 20 μl of bacterial extract and 50 μl of 5 mM *p*-nitrophenyl palmitate (pNPP) were added to the lipase solution. The 20 μl of 30 mM acarbose or 100 μM orlistat (Sigma-Aldrich) served as the positive control in the α-amylase and lipase inhibition assays, respectively. Each sample was reacted at 37°C for 10 min. Thereafter, samples were measured to determine the amount of p-nitrophenol released in the reaction at 410 nm using a UV-vis spectrophotometer (Biotek, USA). The enzyme inhibition rate (%) was expressed as follows in Eq. (2):

$$\text{inhibition rate \%=}\left(\frac{{\text{A}}_{\text{0}}{\text{-A}}_{\text{t}}}{{\text{A}}_{\text{0}}}\right)\text{×100,}$$ (2)

where A_t_ represents the absorbance at time (t) = 10 min and A_0_ is the absorbance at t = 0 h.

### Genome Sequencing and Genomic Analysis

The complete genome sequencing was performed using the de novo MGI platform (BGI, China), according to previous studies [25, 26]. The reads for sequencing were quality trimmed to the Q_30_ confidence level and assembled with CLC Assembly Cell 5.1.1 (Qiagen Inc, USA). The resulting sequences were deposited in GenBank (accession numbers CP104753 and CP104754). The complete genomes were annotated by Rapid Annotation using Subsystem Technology 2.0 (RAST; https://rast.nmpdr.org, RRID:SCR_014606). The genetic circular maps were made using Proksee (https://proksee.ca, RRID:SCR_011779). Average nucleotide identity (ANI) values, digital DNA–DNA hybridization (dDDH) and G+C contents were calculated with the OrthoANI tool (https://www.ezbiocloud.net, RRID:SCR_022562). The functional metabolite biosynthetic gene clusters were analyzed with the antiSMASH v5.1.0 software (https://antismash.secondarymetabolites.org, RRID:SCR_022060).

### Animals, Diets, and Experimental Design

The animal study was carried out in accordance with the ‘Guide for the Care and Use of Laboratory Animals’ as promulgated by the National Institutes of Health, and the protocols were approved by the Ethics Committee of Laboratory Animals at Pukyong National University in Busan, Korea (Approval No: PKNUIACUC-2021-41). Six-week-old specific pathogen-free (SPF; 20210924 Samtako QC) grade C57BL/6J male mice (*n* = 40, 20 ± 2 g) were adopted from Samtako Bio Korea Co., Ltd. (Korea). The mice were housed at 22 ± 2°C and 55 ± 5% humidity under a 12 h diurnal light cycle. In this study, the *Lc. rhamnosus* ATCC 53103 strain, reported to have an anti-obesity effect, was used as a probiotic control. After 2 weeks of adaptation, mice were assigned into different groups (*n* = 10 per group): control (normal-fat diet [NFD]), obese (high-fat diet [HFD]), and HFD + probiotic groups (LR or MGEL20154). The control group was fed with a standard chow diet (10% calories from fat, Diet D12450J, Research Diets Inc., USA), while the diet-induced obese groups were fed the HFD (60% calories from fat, Diet 12492, Research Diets Inc.). The LR or 54 group was administered the probiotic *Lc. rhamnosus* ATCC 53103 or *Lp. plantarum* MGEL201054, respectively, at 5 × 10^8^ CFU in 200 μl/mouse/day with a HFD, while the control and HFD groups received only PBS. Dietary strains were prepared by overnight cultivation in MRS broth at 37°C. The resulting cell pellets were washed twice with PBS and orally supplemented using a gastric tube. The oral administration was maintained for 8 weeks, while regularly controlling body weight, feed, and water intake. Each animal’s body weight was measured weekly, and feed uptake was examined once every 2 days. The feed efficiency (%) was expressed as total body weight gained from the diet divided by total diet consumed during the animal experiments. Weight gain (%) was expressed as follows in Eq. (3):

$$\text{weight gain \%}=\frac{\text{final body weight -initial body weight}}{\text{initial body weigh}}\times 100.$$ (3)

### Histological Analysis

Samples of epididymal fat tissue were removed from each group, fixed in 4% PFA, and embedded in paraffin. Then, 5-μm-thick sections were taken and stained with hematoxylin and eosin. The morphology of the sections was observed under a microscope (Nikon Eclipse 80i, Nikon Co., Japan). Adipocyte size was measured and analyzed using Fiji imaging software with the Adiposoft v1.16 plugin.

### Gene Expression Analysis

For the mRNA expression analysis in Caco-2 cells, cells were diluted in fresh MEM without FBS and streptomycin/penicillin. Caco-2 cells were then seeded in 6-well tissue culture plates (Costar, Corning Inc., USA) at 1 × 10^6^ cells/well. When the cells reached about 75% confluence, they were either treated for 24 h with 100 μg/ml LPS, or, were first exposed to only LPS for 12 h, and then 10^6^ CFU/ml bacterial cells were added for another 12 h together with LPS. The gene expression level was measured by real-time quantitative PCR (RT-qPCR) [22]. After treatment, total RNA was isolated using the Riboclear Plus Kit (GeneAll Biotechnology, Korea). Thereafter, cDNA was synthesized from the isolated RNA as a template using the PrimeScript cDNA Synthesis Kit (TaKaRa Bio, Japan). Gene expression was examined using TB Green Premix Ex Taq (TaKaRa Bio.) on a TP_700/760_ Thermal Cycler Dice (TCD) Real-Time System (Takara). The levels of relative expression were analyzed using the TCD software 5.0 with the 2^-ΔΔCT^ method and GAPDH as a reference gene [27]. The gene-specific primers used for gene amplification are summarized in Table 2.

### Statistical Analysis

All data were analyzed by one-way analysis of variance (ANOVA) using Statistical Package for the Social Sciences (SPSS) followed by Duncan's multiple range test. Statistical significance was accepted at *p* < 0.05 unless otherwise noted.

## Results

### Isolation and Identification of *Lp. plantarum*

For the isolation of *Lp. plantarum*, about 500 colonies were morphologically picked from different kimchi samples. Of these, a total of 270 related LAB strains belonging to three families and 10 genera were identified as *Lactiplantibacillus* spp., *Lacticaseibacillus* spp., *Latilactobacillus* spp., *Lactobacillus* spp., *Leuconostoc* spp., *Pediococcus* spp., *Weissella* spp., *Lactococcus* spp., *Streptococcus* spp., and *Enterococcus* spp., according to the 16S rRNA sequences, which showed 98.9–100% similarity with the corresponding type strains. Finally, the nine selected strains were identified as *Lp. plantarum*; these strains were members of the *Lp. plantarum* subsp. *plantarum* strain and shared the highest sequence similarity with *Lp. plantarum* DSM 20174^T^ (Fig. S1).

### Adhesion Properties of the Isolates

**Fig. 1:**
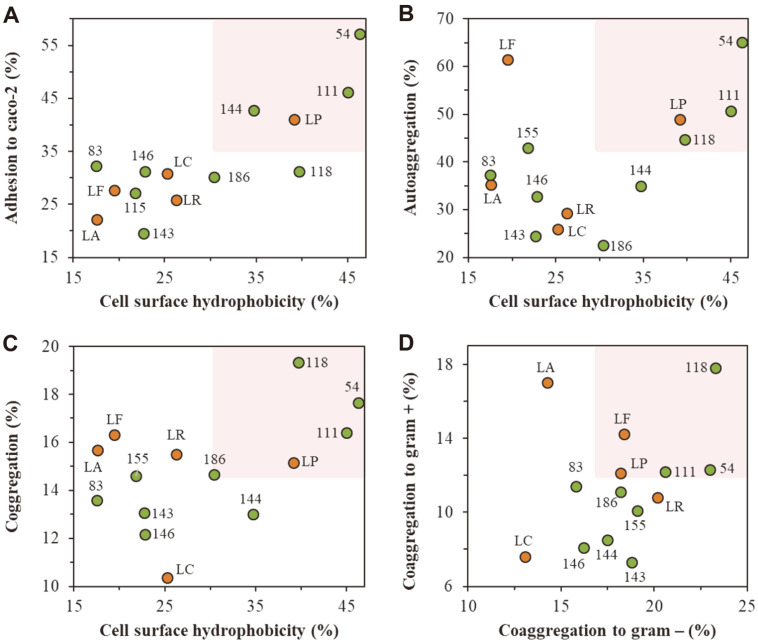
Bacterial cell surface hydrophobicity, autoaggregation, coaggregation, and adhesion to Caco-2 cells. Sections **A, B**, and **C** show the relationship between surface hydrophobicity and Caco-2 cell adhesion, autoaggregation, and coaggregation of the strain, respectively, and **D** shows the coaggregation relationship between gram-negative and grampositive bacteria. LA; *L. acidophilus* ATCC 4356^T^, LC; *Lc. casei* ATCC 393, LF; *Lm. fermentum* IFO 3956^T^, LR; *Lc. rhamnosus* ATCC 53103, LP; *Lp. plantarum* DSM 20174^T^, 54; *Lp. plantarum* MGEL20154, 83; *Lp. plantarum* MGEL21083, 111; *Lp. plantarum* MGEL21111, 118; *Lp. plantarum* MGEL21118, 143; *Lp. plantarum* MGEL21143, 144; *Lp. plantarum* MGEL21144, 146; *Lp. plantarum* MGEL21146, 155; *Lp. plantarum* MGEL21155, and 186; *Lp. plantarum* MGEL21186.

The strains MGEL20154, MGEL21111, and MGEL21118 exhibited strong cell surface hydrophobicity with an adherence of 46.4, 45.0, and 39.7% to hexadecane, respectively (Fig. 1A). Moreover, it was confirmed that strains with high hydrophobicity tend to adhere well to the Caco-2 cell monolayer and are positively related to auto- and coaggregation abilities (Figs. 1B and 1C). On the other hand, no significant correlation was found in the coaggregation between the gram-negative and gram-positive strains; however, similar to cell surface hydrophobicity, the MGEL20154, MGEL21111, and MGEL21118 strains showed better coaggregation abilities in both the gram-negative and gram-positive strains (Fig. 1D).

### Resistance of the Isolates to a Simulated GI Tract

The resistance of the isolates during the in vitro GI condition is shown in Table 3. Although all strains maintained a viable cell count similar to that of the initial inoculation cell count in the saliva phase, a survivability of less than 30% was shown, except for strain MGEL21118 (31.3%), after 3 h in the simulated gastric phase. In the simulated intestinal phase, all tested isolates, except for strains MGEL20154 (59.4%) and MGEL21118 (55.1%), had a survival rate of less than 20%. As a result, the final survivability of the isolates during the simulated GI tract was 17.2% for MGEL21118, followed by 8.8% for MGEL20154, while the other isolates showed a final survival rate of less than 3%. Meanwhile, among the reference strains, the final survivability was less than 5% except for *L. acidophilus* ATCC 4356^T^, which had a final survivability of 12.8%.

### Enzyme Inhibitory Activity of the Selected Isolates

**Fig. 2:**
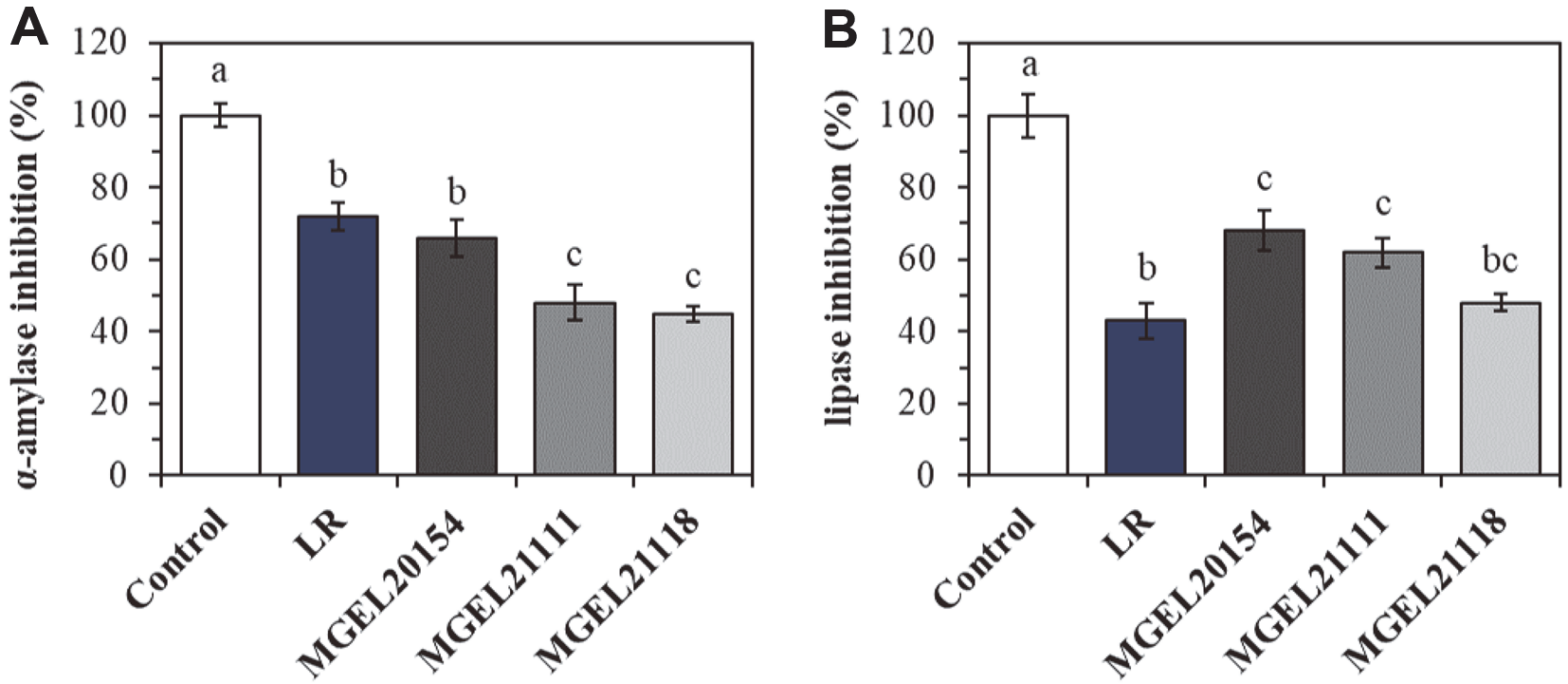
Enzyme inhibition effects. A, B. α-amylase (**A**) and lipase (**B**) inhibition rate. The data are represented as the means ± SD of 10 replicates (10 mice/replicate); means that do not share the same letter differ significantly (*p* < 0.05). LR; *Lc. rhamnosus* ATCC 53103, MGEL20154; *Lp. plantarum* MGEL20154, MGEL21111; *Lp. plantarum* MGEL21111, MGEL21118; *Lp. plantarum* MGEL21118.

The enzyme inhibitory activities of each isolate are shown in Fig. 2. The α-amylase inhibitory activity of MGEL20154 had a significantly higher value (66.8 ± 5.2%) than those of isolates MGEL21111 (48.3 ± 4.8%) and MGEL21118 (45.5 ± 2.1%) (Fig. 2A). And, the inhibition rate of MGEL20154 was not significantly different from that of LR used as a bacterial control. Meanwhile, in pancreatic lipase inhibition, MGEL20154 exhibited significantly higher inhibition of pancreatic lipase (68.1 ± 5.6%) than that of LR (43.7 ± 4.9%) (Fig. 2B). Strains MGEL20154, MGEL21111, and MGEL21118 were initially selected based on intestinal adhesion and GI viability, and strain MGEL20154 was finally selected based on its ability to inhibit α-amylase and lipase activity.

### Comparative Genome Analysis of MGEL20154

**Fig. 3:**
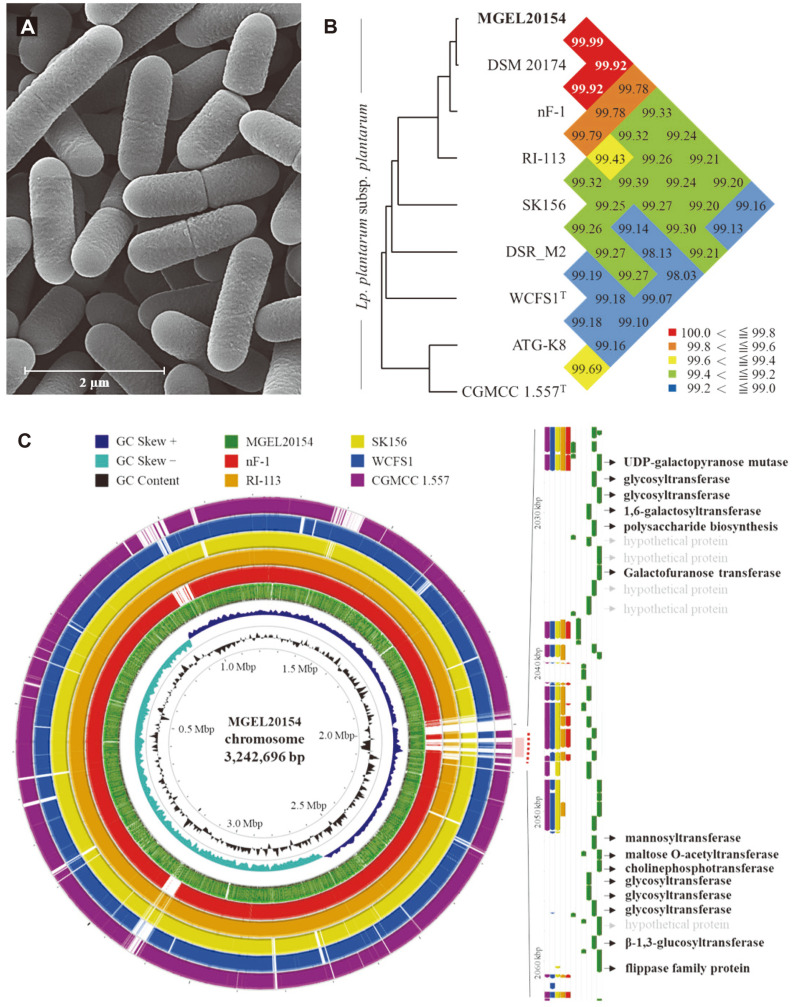
SEM image, ANI, and circular plot of MGEL20154. **A**. SEM of strain MGEL20154 grown on MRS medium for 16 h at 28°C. Bar, 2 μm, **B**. OrthoANI analysis with other related strains, and **C**. Circular plot of the comparison between genomes of MGEL20154 with the closely related strains. From the center to the outside: the number of bases, GC skew (- and +), GC content, location of all annotated open reading frames, *Lp. plantarum* MGEL20154 (MGEL20154), *Lp. plantarum* nF-1 (nF-1), *Lp. plantarum* RI-113 (RI-113), *Lp. plantarum* SK156 (SK156), *Lp. plantarum* WCFS1^T^ (WCFS1^T^) and *Lp. plantarum* CGMCC 1.557^T^ (CGMCC 1.557^T^).

Based on its probiotic properties, isolate MGEL20154 was selected for whole- genome sequencing and subsequent characterization. The complete genome of MGEL20154 consists of a circular chromosome of 3,242,696 bp with one plasmid of 7,221 bp in length, 3,157 coding sequences (CDS) with an average gene length of 866 bp, 16S rRNAs, and 68 tRNAs (Table S1) (accession no. CP104753). The plasmid contained a total of nine CDS consisting of five prophage-related proteins, one cold shock protein, and three hypothetical proteins (Fig. S2). The DNA G+C contents for the chromosome and plasmid were determined to be 44.52 and 36.75%, respectively. Cells of strain MGEL20154 were found to be rod-shaped, 1.2–2.0 μm long, and 0.6–0.7 μm wide using SEM analysis (Fig. 3A). The highest ANI value between the MGEL20154 strain and related strains was 99.98% with DSM 20174^T^ (Accession No. GCA_014131735.1), which was consistent with the result of the phylogenetic tree, followed by 99.92, 99.78, and 99.33% with nF-1 (GCA_003325395.1), RI-113 (GCA_001990145.1), and SK156(GCA_014041895.1), respectively (Fig. 3B). On the other hand, among the *Lp. plantarum* strain types, MGEL20154 was found to have 99.21 and 99.16% identity with WCFS1^T^ (GCA_000203855.3) and CGMCC 1.557^T^ (GCA_001272315.2), respectively. Fig. 3C shows the comparative genomic circular map with closely related members of the strains. Interestingly, the MGEL20154 strain was found to have many glycosyltransferases in two specific loci between the genome positions at 2,030 kbp and 2,060 kbp. These glycosyltransferase genes were analyzed and found to be a characteristic of only MGEL20154, except for the genetically closest strain, DSM 20174^T^. Furthermore, based on the genome analysis on the RAST server, around 25% of detected genes - a total of 722 genes—were annotated in the subsystem (Fig. S3). The genes associated with carbohydrate (231), amino acid (171), and protein (134) metabolism have been mostly identified. The antiSMASH results on the functional metabolites showed the plantaricin biosynthesis gene cluster had 85-100% of gene sequence similarity with several *Lp. plantarum* and *Lp. paraplantarum* strains (data not shown).

### Anti-Obesity Effects of MGEL20154 in HFD-Induced Obese Mice

**Fig. 4:**
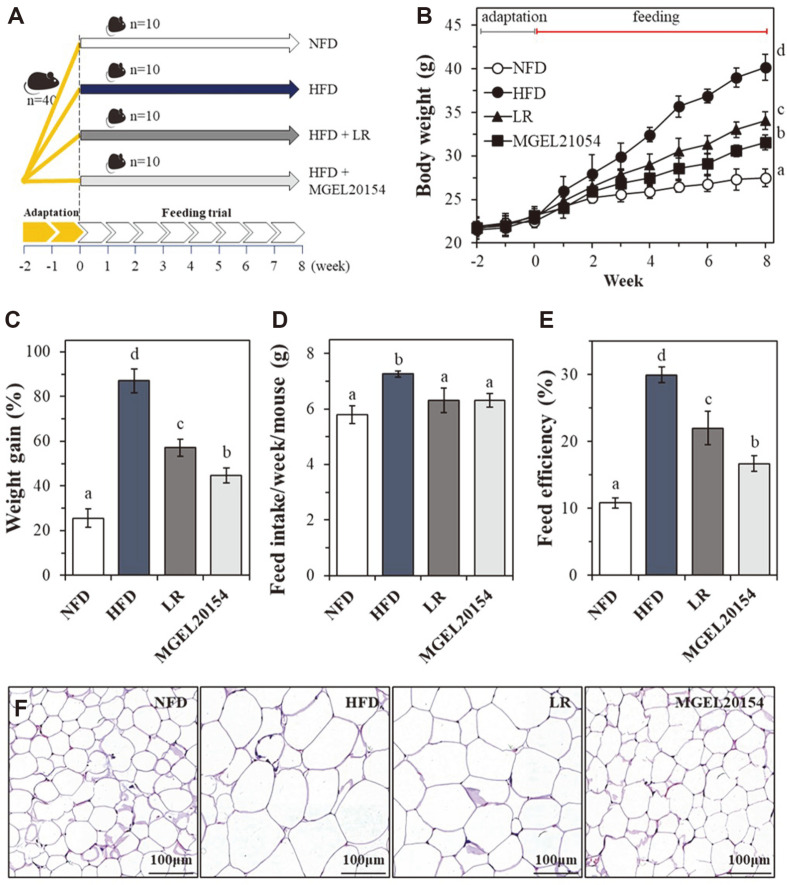
Effects of MGEL20154 administration in C57BL/6J obese mice. **A**. scheme of experiment design, **B**. body weight evolution, **C**. weight gain (%), **D**. feed intake/week/mouse (g), **E**. feed efficiency, and **F**. epididymal adipocyte size of diet-induced obese mice. The data are represented as the means ± SD of 10 replicates (10 mice/replicate); means that do not share the same letter differ significantly (*p* < 0.05). NFD; nomal fat diet-fed group, HFD; high-fat diet-fed group, LR; HFD-fed *Lc. rhamnosus* ATCC 53103-treated group, MGEL20154; HFD-fed *Lp. plantarum* MGEL20154-treated group.

Four different diets, NFD, HFD, HFD+LR, and HFD+MGEL20154, were orally administered to C57BL/6J mice for 8 weeks except for 2 weeks of NFD feeding for the adaptation period, and body weight and feed intake were measured daily (Figs. 4A-4E). HFD feeding resulted in significant weight gain compared to that of the other three groups from 1 week after starting each diet (Fig. 4B). After 8 weeks, mice fed the HFD showed an average body weight of 40.2 ± 1.4 g and a weight gain of 86.9 ± 5.3%, resulting in a strong increase in body mass (NFD; 27.5 ± 0.9 g and 25.6 ± 4.1%) (Fig. 4C). HFD + probiotic (LR or MGEL20154) groups showed a significantly lower weight increase compared to that of the HFD group. There was a significant reduction in weight gain at 3 weeks for the HFD + MGEL20154 group and 4 weeks for the HFD + LR group, compared to that of the HFD group. After the test period, the HFD-MGEL20154 and HFD-LR groups exhibited a body weight of 34.1 ± 1.0 g and 31.6 ± 0.8 g, respectively, and a weight gain of 57.1 ± 3.8% and 44.8 ± 3.2%, respectively, showing significantly reduced weight gain compared to that of the HFD group. In addition, strain MGEL20154 showed a stronger weight gain reduction effect than was seen in the HFD-LR group. Although, there was no significant difference in the weekly dietary intake except for the HFD-group (NFD; 5.8 ± 0.3 g, HFD; 7.3 ± 0.1 g, HFD + LR; 6.3 ± 0.4 g, and HFD + MGEL20154; 6.3 ± 0.2 g), the feed efficiency in the HFD + MGEL20154 group was significantly lower than that in the other groups except for the NFD-group (NFD; 10.8 ± 0.7%, HFD; 30.0 ± 1.1%, HFD+LR; 22.1 ± 2.5%, and HFD + MGEL20154; 16.6 ± 1.1%) (Figs. 4D and 4E). Histological examination revealed that the average epididymal adipocyte size in the HFD group was markedly increased relative to that of the NFD or HFD + probiotic adipocytes (Fig. 4F). Meanwhile, quantitative assessment of adipocyte size clearly demonstrated that the HFD + MGEL20154 group presented a smaller adipocyte area (1785 ± 78 μm^2^) compared to that of the HFD (2387± 89 μm^2^), HFD + LR (2165 ± 93 μm^2^), and NFD (1533 ± 31 μm^2^) groups.

### Regulation of Gene Expression in Caco-2 Cells by MGEL20154

**Fig. 5:**
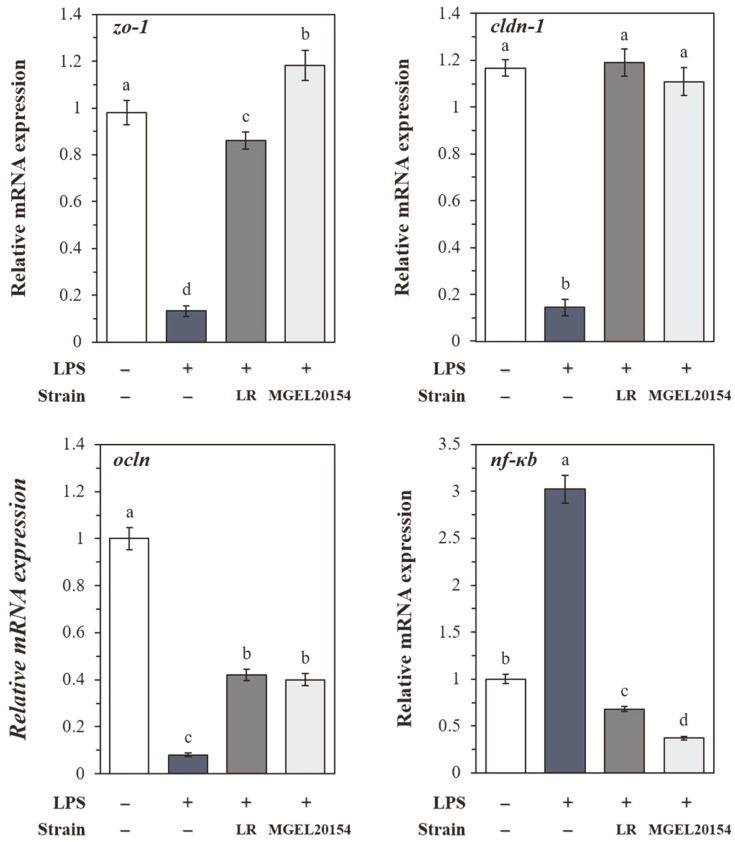
The effect of MGEL20154 treatment on the mRNA expression of *zo-1*, *cldn-1*, *ocln*, and *nf-κb* in LPS-induced Caco-2 cells. The data are represented as the means ± SD of 10 replicates (10 mice/replicate); means that do not share the same letter differ significantly (*p* < 0.05). LR; HFD-fed *Lc. rhamnosus* ATCC 53103-treated group, MGEL20154; HFD-fed *Lp. plantarum* MGEL20154-treated group.

**Fig. 6:**
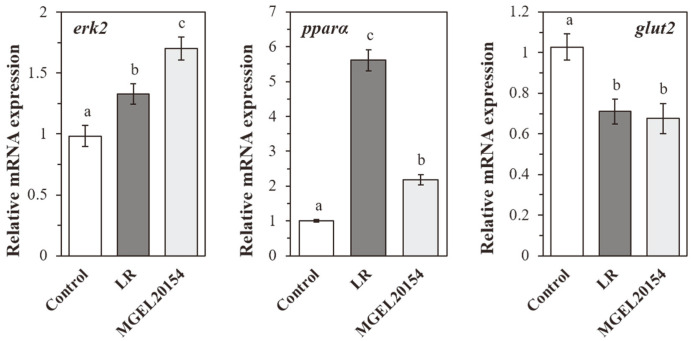
The effect of MGEL20154 treatment on the mRNA expression of *erk2*, *pparα*, and *glut2* in Caco-2 cells treated with MGEL20154, respectively. The data are represented as the means ± SD of 10 replicates (10 mice/replicate); means that do not share the same letter differ significantly (*p* < 0.05). LR; HFD-fed *Lc. rhamnosus* ATCC 53103-treated group, MGEL20154; HFD-fed *Lp. plantarum* MGEL20154-treated group.

The LPS-induced damage in Caco-2 cells was clearly demonstrated by decreasing the expression of the *zo-1*, *cldn-1*, and *ocln* genes involved in epithelial barrier integrity and increasing the expression of the inflammatory *nf-ĸb* (Fig. 5). Meanwhile, the mRNA expression of *zo-1*, *cldn-1*, and *ocln* was rapidly restored in the MGEL20154-treated group (Fig. 5). In particular, 12 h of MGEL20154 treatment showed an 18% increase in *zo-1*, *cldn-1*, and *ocln* expression levels compared to that of normal cells, and the gene expression recovery of *cldn-1* and *ocln* was 82% and 42%, respectively, compared to that of normal cells (*p* < 0.05). No statistically significant differences except for *zo-1* were observed in the mRNA expression of both *cldn-1* and *ocln* between the LR (*cldn-1* or *ocln/gapdh*; 1.02 and 0.42) and MGEL20154 (0.95 and 0.40) groups (*p* < 0.05). In addition, the gene expression of *nf-ĸb*, which increased nearly three folds (*nf-ĸb*/*gapdh*; 3.02) due to the inflammatory response induced by LPS, was significantly downregulated after MGEL20154 treatment, showing an expression level of 37% compared to that of normal cells (*p* < 0.05, Fig. 5). Fig. 6 shows the regulation of *erk2*, *pparα*, and *glut2* mRNA in the MGEL20154-treated Caco-2 cell monolayer. In the MGEL20154-treated group, *erk2* and *pparα* were significantly upregulated, and *pparα* was downregulated compared to that of normal cells (*p* < 0.05). Although there was a significant difference at the transcriptional level, both MGEL20154- and LR-treated groups showed similar gene regulation tendencies for the three genes.

## Discussion

Probiotics are known to be closely linked to all aspects of human health and disease by interacting with epithelial cells in the host digestive tract, resulting in modulated homeostasis, such as in the immune system, metabolism, and mental health [28-30]. Currently, probiotics are used as microbiome drugs to treat diseases by modulating the host microbiome, through lowering of the pH in the digestive tract, enhancing barrier motility, and inhibiting pathogenic microbes [31]. To exert these health benefits, intestinal adhesion that lasts for a certain time period is important. However, since many probiotics are still limited in exerting enough physiological functions due to insufficient adhesion and colonization, in-depth studies are being conducted to overcome this dilemma [32]. It has been reported that various factors are involved in cell adhesion. Summary of the literature has shown, however, that probiotic adhesion might be comprehensively involved with non-specific factors related to bacterial cell surface hydrophobicity, such as s-layers, peptidoglycans, phospholipids, glycoproteins, and oligo-and polysaccharides, rather than the specific factors that cell surface proteins bind to on the mucosal layer [33, 34]. Consistent with previous studies, the cell surface hydrophobicity of the isolates in this study was found to be positively correlated with adhesion to Caco-2 cells.

Glycosyltransferases are essential enzymes for the synthesis of di-, oligo-, and polysaccharides, glycans, and glycoproteins, which are considered as the major macromolecules that determine microbial-host interactions [35]. Although the elucidation of their exact role is complex since most bacteria contain more than one type of surface polysaccharide, an important biological role for these macromolecules has recently been shown in gut colonization [36-38]. Furthermore, gut microbes participate in carbohydrate metabolism by expressing glycosyltransferases to join carbohydrates [39]. MGEL20154 can exert an anti-obesity effect by consuming the carbon source of the host for its own benefit. Consistent with previous studies, the abundance of genes involved in the carbohydrate metabolic pathway of the microbiome can be inferred as positively correlated with the anti-obesity effect [40, 41]. Strain MGEL20154 was expected to maximize the unique health benefits of the *Lp. plantarum* strain because it can promote adhesion and colonization due to its strong survival rate in the GI tract and the hydrophobicity of the bacterial cell surface. In addition, based on whole-genome analysis and the results of the strain-fed animal experiment, the presence of various carbohydrate utilization genes in MGEL20154 suggests it has a reliable potential for decreasing weight gain by significantly reducing feed efficiency by calorie restriction along with aggressive carbohydrate consumption in the gut of mice, resulting in the mitigation of increased adipocyte size.

In the analysis of gene expression patterns in MGEL20154-treated Caco-2 cells, MGEL20154 treatment significantly restored LPS-induced damage in the Caco-2 monolayer. In addition, it was confirmed that the inflammatory response induced by the endotoxin can be alleviated through downregulation of *nf-ĸb*. This suggests that there is a strong possibility to alleviate obesity and metabolic diseases mediated by endotoxin-induced leaky gut and inflammation due to the dysbiosis of intestinal microflora following a high-fat diet. Numerous studies have reported a correlation between the high-fat diet and the hyperpermeability of the intestinal barrier that results in metabolic disorders [42-44]. There is well established evidence that a high-fat diet leads to a decrease in microbial diversity and a dysbiosis of microbiota between Firmicutes and Bacteroidetes, which causes leaky gut and results in inflammatory bowel disease (IBD) [45]. In addition, IBD patients often present with several extraintestinal manifestations that can be caused by chronic inflammation, and these comorbidities include metabolic syndromes, which can eventually lead to obesity [46]. However, the anti-obesity mechanism of MGEL20154 has been demonstrated to restore intestinal barrier integrity and modulate the immune response. A potential internal mechanism of barrier damage repair by MGEL20154 may be associated with inhibition of nf-κb expression and activation of mitogen-activated protein kinase (*mapk*)-*erk2*. It is reported that the overexpression of *NF-κB* could facilitate the expression of myosin light chain (MLC) kinase, which is regarded to promote tight junction permeabilization by catalyzing the phosphorylation of MLC proteins, thus increasing barrier permeability under inflammatory conditions [47]. Moreover, ERKs in the MAPK pathway were shown to have a protective role on tight junctions in cell monolayers [48]. Consistent with previous studies, the present study also demonstrated downregulated nf-κb and upregulated *mapk/erk* expression in the MGEL20154-treated Caco-2 cells, suggesting this might be the potential mechanism of the protective function of MGEL20154 in the intestinal epithelial barrier.

PPARs, which comprise PPARα, PPARγ, and PPARβ/δ, are ligand-activated transcription factors of the nuclear hormone receptor superfamily. They are mostly found in the intestinal epithelium and the isoform PPARγ plays a key role in fatty acid metabolism regulation through β-oxidation, cell proliferation, and intestinal homeostasis [49]. Although PPARα is also a major regulator of lipid metabolism controlled by free fatty acids (particularly in the liver), its role and importance in intestinal epithelial cells has been less studied than that of PPARγ due to its relatively low expression in the liver. However, it has been reported that the upregulated expression level of PPARα in the human intestine plays a role in the reduction of intestinal inflammation and decreased intestinal permeability as well as reduced inflammation caused by ischemia and reperfusion [50-52]. Upregulated PPARα expression in Caco-2 cells induced by specific probiotics is reported to inhibit Niemann-Pick C1-like intracellular cholesterol transporter 1 (NPC1L1) expression, thereby lowering the cellular cholesterol content [53-55]. In addition, *Lc. rhamnosus* strain GG could suppress NPC1L1 expression in Caco-2 cells, and in our study, we confirmed that LR acts as a potent PPARα activator [56]. These results support the hypothesis of the previous study that transcriptional regulation of PPARα may eventually inhibit cholesterol absorption in the body and thus alleviate hyperlipidemia and obesity [57, 58]. Therefore, MGEL20154, also a potent activator of *pparα*, could inhibit NPC1L1 expression in Caco-2 cells, suggesting that it can suppress cholesterol absorption into cells from the digestive tract and result in an anti-obesity effect.

The intestines have a determinant role in energy homeostasis to ensure food digestion, nutrient absorption, and gut-hormone release in response to dietary compounds. In general, the flow of absorbed sugars into the digestive tract, enterocytes, and blood depends on the surface membrane transporters of enterocytes. In epithelial cells, sodium-glucose linked transporter 1 (SGLT1) promotes the uptake of sugar in the intestine, and GLUT-2 mediates the flux of sugars from epithelial cells across the basolateral membrane to the blood [59]. The insulin resistance accompanied by obesity causes a disturbance of GLUT-2 trafficking control, leading to permanent localization of GLUT-2 in the apical and endosomal enterocyte membranes. This in turn increases transepithelial glucose transport from the lumen to the bloodstream [60, 61]. Eventually, the blood glucose concentration increases, and glucose is absorbed into adipocytes, which further triggers obesity and causes comorbidities, such as metabolic diseases. Therefore, direct inhibition of GLUT-2 as the main glucose transporter in the enterocyte could significantly regulate glucose ingestion from the intestine [62-64]. Our results showed that MGEL20154 could significantly downregulate the transcription of GLUT-2 in Caco-2 cells, suggesting GLUT-2 modulation through MGEL20154 intake may be a mechanism of weight gain reduction.

**Fig. 7:**
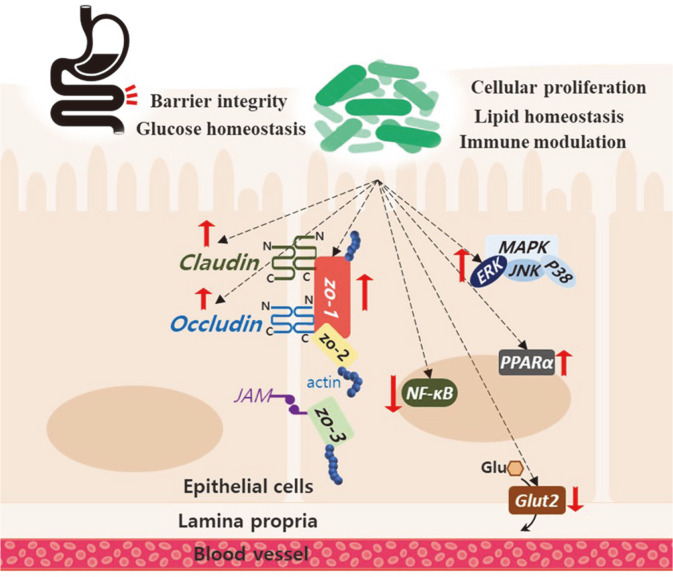
Proposed mechanism of *Lp. plantarum* MGEL20154 on anti-obesity effects through regulating mRNA expression in epithelial cells.

In summary, three strains isolated from kimchi, MGEL20154, MGEL21111, and MGEL21118, showed excellent adhesion abilities and survival rates in the GI tract out of 14 strains, including four type strains and nine isolates. Among these, the MGEL20154 strain was finally selected based on its inhibition ability against both α-amylase and lipase activity. Genome analysis showed MGEL20154 had many glycosyltransferases, suggesting an anti-obesity effect occurs through calorie restriction. Oral administration of MGEL20154 to diet-induced obese C57BL/6 mice resulted in a 44.7% reduction in feed efficacy compared to that of the HFD group, partially supporting the hypothesis of an anti-obesity effect through calorie restriction. In addition, the reduction rate of weight gain in the HFD + MGEL20154 group was about 48.5% compared to that of the HFD group after 8 weeks, and the epididymal fat pad was also reduced by 25.2%. In the transcription analysis of Caco-2 cells treated with MGEL20154, the expression of *zo-1*, *erk2*, and *pparα* was significantly upregulated, and *nf-ĸb* and *glut2* was significantly downregulated (Fig. 7). However, a limitation of this study is that further research is needed to determine how glycosyltransferase genes play a role in calorie restriction, how the modulation of gene expression in epithelial cells is organically related, and how the microbiome changes. We are only just beginning to understand the interconnections between probiotics, gut microbiome, and host health. Although our picture of close correlation remains incomplete, strain-specific probiotic mechanism studies on their health benefits will lead to a better understanding of their functions as well as microbe and human interactions.

## Supplemental Materials

Supplementary data for this paper are available on-line only at http://jmb.or.kr.

## Figures and Tables

**Table 1:** Bacterial strains used and culture conditions.

Microorganisms	Strain	Temperature	Medium	Atmosphere
Lactic acid bacteria				
*Lactobacillus acidophilus*	ATCC 4356^T^	35°C	MRS	5% CO_2_
*Lacticaseibacillus casei*	ATCC 393	35°C	MRS	5% CO_2_
*Limosilactobacillus fermentum*	KCTC 13097^T^	35°C	MRS	5% CO_2_
Lactiplantibacilus plantarum	DSM 20174^T^	35°C	MRS	5% CO_2_
*Lacticaseibacillus rhamnosus*	ATCC 53103 (GG)	35°C	MRS	5% CO_2_
Lactiplantibacilus plantarum	MGEL20154^†^	35°C	MRS	5% CO_2_
Lactiplantibacilus plantarum	MGEL21083^†^	35°C	MRS	5% CO_2_
Lactiplantibacilus plantarum	MGEL21111^†^	35°C	MRS	5% CO_2_
Lactiplantibacilus plantarum	MGEL21118^†^	35°C	MRS	5% CO_2_
Lactiplantibacilus plantarum	MGEL21143^†^	35°C	MRS	5% CO_2_
Lactiplantibacilus plantarum	MGEL21144^†^	35°C	MRS	5% CO_2_
Lactiplantibacilus plantarum	MGEL21146^†^	35°C	MRS	5% CO_2_
Lactiplantibacilus plantarum	MGEL21155^†^	35°C	MRS	5% CO_2_
Lactiplantibacilus plantarum	MGEL21186^†^	35°C	MRS	5% CO_2_
Indicator				
Gram -				
*Vibrio parahaemolyticus*	ATCC 33844^T^	25°C	BHI	Aerobic
*Shigella sonnei*	MGE2007^†^	35°C	BHI	Aerobic
Gram +				
*Listeria monocytogenes*	KCTC 13064^T^	35°C	BHI	Aerobic
*Bacillus cereus*	KCTC 3624^T^	35°C	BHI	Aerobic

^†^Laboratory collection

**Table 2:** Gene specific primers used in this study.

Gene	Product	Oligonucleotide Sequence (5` to 3`)	Size (bp)	Ref. sequence (Accession No.)
*zo-1*	Zonula occludens-1 (ZO-1)	F TTCACGCAGTTACGAGCAA R TTGGTGTTTGAAGGCAGAG	141	XM_047432991.1
*cldn-1*	Claudin-1 (CLDN-1)	F TGGTCAGGCTCTCTTCACTG R TTGGATAGGGCCTTGGTGTT	119	NM_021101.5
*ocln*	Occludin (OCLN)	F TTGGATAGGGCCTTGGTGTT R GCCTGTAAGGAGGTGGACT	85	NM_001205254.2
*nf-κb*	Nuclear factor κB subunit 1 (NF-κB)	F AGCAAATAGACGAGCTCCG R TCGGTAAAGCTGAGTTTGC	81	NM_001319226.2
*erk2*	Extracellular signal-regulated kinase 2 (ERK2)	F TTCCCTGGTTCTCTCTAAAG R GGGTCTGTTTTCCGAGGATG	184	NM_002745.5
*glut2*	Glucose transporter type 2 (GLUT-2)	F GTTAGATGAGGAAGTCAAA R CCAGCTACCGACAGCCTA	165	NM_000340.2
*pparα*	Peroxisome proliferator activated receptor alpha (PPARα)	F CTGAGCCATGCAGAATTTAC R GTCTAAGGCCTCGCTGGTG	118	NM_001001929.3
*gapdh*	glyceraldehyde-3-phosphate dehydrogenase (GAPDH)	F GATGCTGGCGCTGAGTA R GGCAGAGATGATGACCCT	105	NM_001256799.3

**Table 3:** Acid and bile tolerance of lactic acid bacteria in the simulated gastrointestinal tract.

Gastrointestinal phase	Time (h)	Viable cell count (×10^6^ CFU ml^-1^)													

		Reference strains					Isolates; *Lp. plantarum* strain MGEL series								
	
		LA	LC	LF	LR	LP	54	83	111	118	143	144	146	155	186
Initial CFU	0.0	822	1,325	1,892	3,667	1,350	1,750	1,843	3,435	1,067	2,485	2,267	2,513	3,120	1,950
Saliva phase	0.1	802	1,320	1,888	3,665	1,351	1,750	1,843	3,430	1,067	2,485	2,148	2,511	3,120	1,950
Gastric phase	3.0	305	234	752	367	362	259	267	526	334	348	322	459	522	405
Intestinal phase	5.0	175	162	103	42	212	300	224	326	337	300	457	428	400	385
	7.0	166	75	–	23	98	267	145	267	340	203	567	362	282	158
	9.0	150	50	–	18	85	234	109	186	352	111	167	198	170	62
	11.0	105	46	–	1	68	154	47	70	184	18	13	15	54	7
Acid tolerance^a^		37.1	17.7	39.7	10.0	26.8	14.8	14.4	15.3	31.3	14.0	14.2	18.2	16.7	20.7
Bile tolerance^b^		34.4	19.7	–	0.2	18.7	59.4	17.6	13.3	55.1	5.2	4.0	3.2	10.3	1.7
Survivability^c^		12.8	3.5	–	<0.1	5.0	8.8	2.5	2.0	17.2	0.7	0.6	0.6	1.7	0.3

^a^% = (CFU initial count / CFU at 3 h) × 100; ^b^% = (CFU at 3 h / CFU final count) × 100; ^c^% = (CFU initial count / CFU final count) × 100. Data presented are mean values of the results obtained from triplicate experiments; the standard deviation is < 0.05%. LA; *L. acidophilus* ATCC 4356^T^, LC; *Lc. casei* ATCC 393, LF; *Lm. fermentum* IFO 3956^T^, LR; *Lc. rhamnosus* ATCC 53103, LP; *Lp. plantarum* DSM 20174^T^, 54; *Lp. plantarum* MGEL20154, 83; *Lp. plantarum* MGEL21083, 111; *Lp. plantarum* MGEL21111, 118; *Lp. plantarum* MGEL21118, 143; *Lp. plantarum* MGEL21143, 144; *Lp. plantarum* MGEL21144, 146; *Lp. plantarum* MGEL21146, 155; *Lp. plantarum* MGEL21155, and 186; *Lp. plantarum* MGEL21186.
