# Supplementary material for: Anti-Obesity Potential through Regulation of Carbohydrate Uptake and Gene Expression in Intestinal Epithelial Cells by the Probiotic Lactiplantibacillus plantarum MGEL20154 from Fermented Food
Source: J Microbiol Biotechnol. 2023 Feb 6;33(5):621–33. doi: 10.4014/jmb.2212.12005 (PMC10236178; doi:10.4014/jmb.2212.12005)
Supplement: Supplementary file 1 [file jmb-33-5-621-supple.pdf]

J. Microbiol. Biotechnol.
https://doi.org/10.4014/jmb.2212.12005

Supplementary Figures and Table

Anti-obesity potential through regulation of carbohydrate uptake and gene expression in intestinal epithelial cells by the probiotic, *Lactiplantibacillus plantarum* MGEL20154, derived from fermented food

So Young Park[1], Jin Won Choi[1], Dong Nyoung Oh[1], Eun Ji Lee[1], Dong Pil Kim[1], Sun Jay Yoon[1], Won Je Jang[1], Sang Jun Han[1], Seungjun Lee[2] and Jong Min Lee[1*]

[1]Department of Biotechnology, Pukyong National University, Busan, 48513, Republic of Korea

[2]Department of Food Science and Nutrition, Pukyong National University, Busan, 48513, Republic of Korea.

*Corresponding author:

Jong Min Lee, jmlee84@pknu.ac.kr

Contents

Table S1. General genomic features of the MGEL20154 with related strains. 2

Fig. S1. Sequence identity matrix and neighbor-joining phylogenetic tree of isolates. 3

Fig. S2. Circular plot of the MGEL20154 plasmid. 5

Fig. S3. Genome annotation results of MGEL20154 on the RAST webserver. 6

Table S1. General genomic features of the MGEL20154 and related members of the *Lp. plantarum* subsp. *plantarum*.

Features	MGEL20154	DSM 20174^T	nF-1	RI-113	SK156	WCFS1^T	CGMCC 1.557^T
Chromosome							
Genome size (bp)	3,242,696	3,242,936	3,120,761	3,249,180	3,231,383	3,308,273	3,156,839
Protein coding sequence (no.)	3,157	2,922	2,845	3,169	2,899	3,041	2,913
16S rRNA genes (no.)	5	5	5	5	5	5	5
23S rRNA genes (no.)	5	5	5	5	5	5	5
5S rRNA genes (no.)	6	6	6	6	6	6	6
tRNA genes (no.)	68	71	67	67	67	72	74
Pseudogenes (no.)		47	65	67	25	26	99
Plasmids							
Number	1	1	2	6	-	3	2
bp	7,221	7,218	40,759<br>30,472	51,784<br>43,225<br>37,427<br>27,923<br>17,514<br>35,937	-	1,917<br>2,365<br>36,069	67,395<br>49,005
G+C content (%)	44.52	44.48	44.61	44.34	44.60	44.45	44.43

GenBank assembly accession numbers; DSM 20174, GCA_014131735.1; nF-1, GCA_003325395.1; RI-113, GCA_001990145.1; SK156, GCA_014041895.1; WCFS1^T, GCA_000203855.3; CGMCC 1.557^T, GCA_001272315.2.

a)

	LA	LC	LF	LR	LPEN	LPP	LP	54	83	111	118	143	144	146	155	186
**LA**	ID	87.0	87.4	87.2	86.4	86.5	86.3	86.3	86.3	86.3	86.3	86.3	86.3	86.3	86.3	86.3
**LC**	87.0	ID	89.2	98.5	91.4	91.4	91.4	91.4	91.4	91.4	91.4	91.4	91.4	91.4	91.4	91.4
**LF**	87.4	89.2	ID	89.4	89.7	89.6	89.8	89.8	89.8	89.8	89.8	89.8	89.8	89.8	89.8	89.8
**LR**	87.2	98.5	89.4	ID	92.1	92.0	92.1	92.1	92.1	92.1	92.1	92.1	92.1	92.1	92.1	92.1
**LPEN**	86.4	91.4	89.7	92.1	ID	99.7	99.9	99.9	99.9	99.9	99.9	99.9	99.9	99.9	99.9	99.9
**LPP**	86.5	91.4	89.6	92.0	99.7	ID	99.6	99.6	99.6	99.6	99.6	99.6	99.6	99.6	99.6	99.6
**LP**	86.3	91.4	89.8	92.1	99.9	99.6	ID	100	100	100	100	100	100	100	100	100
**54**	86.3	91.4	89.8	92.1	99.9	99.6	100	ID	100	100	100	100	100	100	100	100
**83**	86.3	91.4	89.8	92.1	99.9	99.6	100	100	ID	100	100	100	100	100	100	100
**111**	86.3	91.4	89.8	92.1	99.9	99.6	100	100	100	ID	100	100	100	100	100	100
**118**	86.3	91.4	89.8	92.1	99.9	99.6	100	100	100	100	ID	100	100	100	100	100
**143**	86.3	91.4	89.8	92.1	99.9	99.6	100	100	100	100	100	ID	100	100	100	100
**144**	86.3	91.4	89.8	92.1	99.9	99.6	100	100	100	100	100	100	ID	100	100	100
**146**	86.3	91.4	89.8	92.1	99.9	99.6	100	100	100	100	100	100	100	ID	100	100
**155**	86.3	91.4	89.8	92.1	99.9	99.6	100	100	100	100	100	100	100	100	ID	100
**186**	86.3	91.4	89.8	92.1	99.9	99.6	100	100	100	100	100	100	100	100	100	ID

100.0   100.0 < ≦ 99.8   99.8 < ≦ 99.6   99.6 < ≦ 98.0   98.0 < ≦ 90.0   90.0 < ≦ 85.0

b)

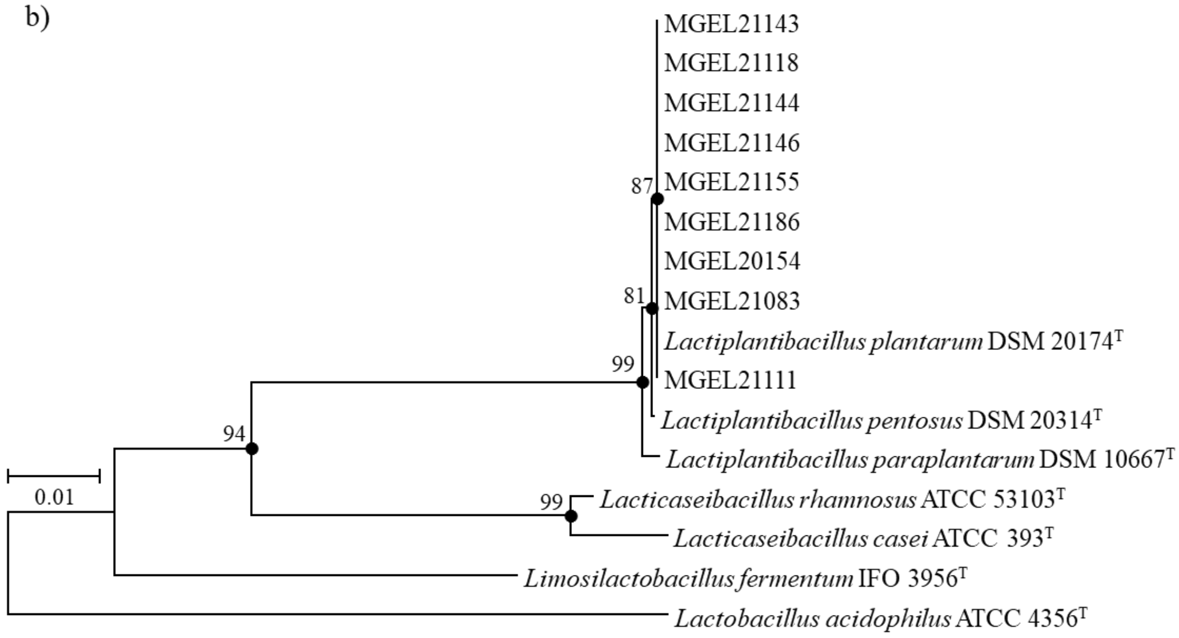

Fig. S1. Sequence identity matrix and neighbor-joining phylogenetic tree of isolates. (a) Sequence identity matrix (%) of 16S rRNA sequences from seven reference probiotics and nine isolates. The boxes are shaded in different colors according to similarity. LA; *Lactobacillus acidophilus* ATCC 4356^T (accession number; GCA_003047065.1), LC; *Lacticaseibacillus casei* ATCC 393^T (GCA_000829055.1), LF; *Limosilactobacillus fermentum* IFO 3956^T (GCA_000010145.1), LR; *Lacticaseibacillus rhamnosus* ATCC 53103^T (GCA_000026505.1), LPEN; *Lactiplantibacillus pentosus* DSM 20314^T (GCA_003641185.1), LPP; *Lactiplantibacillus paraplantarum* DSM 10667^T (GCA_003641145.1), LP; *Lactiplantibacillus*

plantarum DSM 20174^T (GCA_014131735.1), 54; *Lactiplantibacillus plantarum* MGEL20154, 83; *Lactiplantibacillus plantarum* MGEL21083, 111; *Lactiplantibacillus plantarum* MGEL21111, 118; *Lactiplantibacillus plantarum* MGEL21118, 143; *Lactiplantibacillus plantarum* MGEL21143, 144; *Lactiplantibacillus plantarum* MGEL21144, 146; *Lactiplantibacillus plantarum* MGEL21146, 155; *Lactiplantibacillus plantarum* MGEL21155, and 186; *Lactiplantibacillus plantarum* MGEL21186. (b) Neighbor-joining phylogenetic tree based on 16S rRNA gene sequences showing the relationships of nine isolate strains with *Lactiplantibacillus plantarum* DSM 20174^T and other members of the probiotic strains. Bootstrap percentages (50%) based on 1000 resamplings are given at the nodes. Dots indicate branches that were also recovered by using maximum-parsimony and maximum-likelihood algorithms. Bar, 0.01 substitutions per nucleotide position.

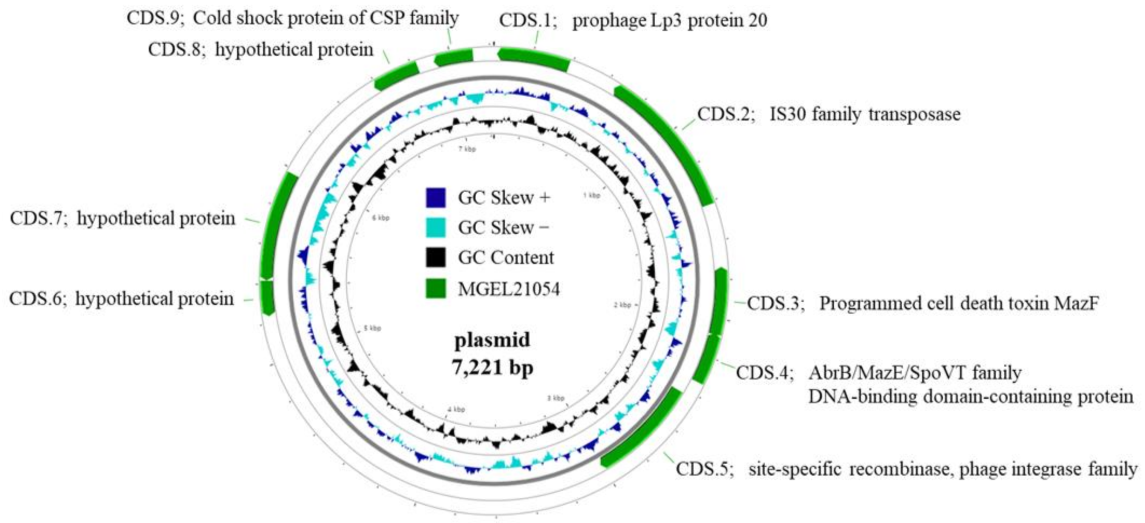

Fig. S2. Circular plot of the MGEL20154 plasmid.

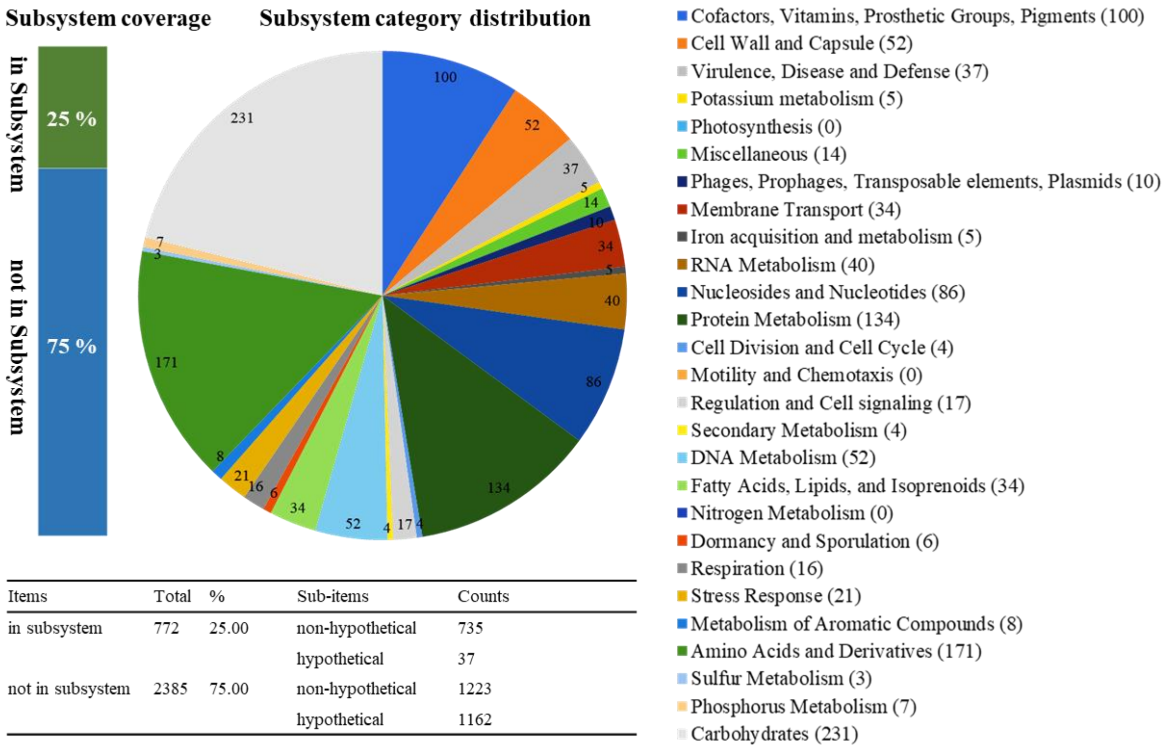

Items	Total	%	Sub-items	Counts
in subsystem	772	25.00	non-hypothetical	735
			hypothetical	37
not in subsystem	2385	75.00	non-hypothetical	1223
			hypothetical	1162

Fig. S3. Genome annotation results of MGEL20154 on the RAST webserver.
